# Assessing the performance of a disposable electrochemical biofilm test kit on monitoring drainage sludge biofilm corrosion and its biocide treatment

**DOI:** 10.1007/s00449-025-03173-x

**Published:** 2025-05-05

**Authors:** Lingjun Xu, Chris Gu, Shaohua Wang

**Affiliations:** 1 Department of Chemical and Biomolecular Engineering, Institute for Corrosion and Multiphase Technology, Ohio University, Athens, 45701 USA; 2 Department of Biomedical Sciences, Ohio University Heritage College of Osteopathic Medicine, Ohio University, Athens, OH 45701 USA; 3 Infectious and Tropical Disease Institute, Ohio University, Athens, OH 45701 USA

**Keywords:** Bioelectrochemistry, Microbiologically influenced corrosion, Carbon steel, Sludge biofilm, Biocide

## Abstract

Drainage sludge is abundant with corrosive microbes which can contribute to soil corrosion of buried pipelines. In this work, the microbiologically influenced corrosion (MIC) of a drainage sludge biofilm against X65 carbon steel was confirmed by significant uniform corrosion (0.03 mm/a uniform corrosion rate) and more severe pitting corrosion (18% greater) on X65 coupons with nutrient enrichment without venting at 37 ℃ compared to the aerobic sludge at room temperature. A new biofilm/MIC test kit was employed to assess the aerobic sludge biofilm, and it was determined to be mildly corrosive against carbon steel after incubating 5 mL of aerobic sludge at room temperature for 7 d in the 10 mL biofilm test kit vial. Tetrakis-hydroxymethyl phosphonium sulfate (THPS), a green biocide was also tested in the biofilm/MIC test kit for its mitigation of the aerobic sludge biofilm and its corrosion against the X65 carbon steel working electrode. The biofilm test kit successfully monitored the sludge biofilm’s sanitization efficacy. It was found that 100 ppm THPS was effective in inhibiting biofilm growth, and 400 ppm THPS in treating pre-established sludge biofilm by achieving 10% corrosion rate reduction. Thus, the biofilm/MIC test kit was found to be sensitive in detecting MIC and can be used as a convenient tool in assessing biofilm corrosivity and its mitigation efficacy.

**Supplementary Information:**

The online version contains supplementary material available at 10.1007/s00449-025-03173-x.

## Introduction

Corrosion is the deterioration of steel which can eventually lead to infrastructure failures [1, 2]. Metallic pipelines and tanks buried in soil suffer from corrosion through direct contact with the external aggressive environment, which is a major problem in water, sewer, and oil and gas distribution systems [3–5]. Previous studies have identified corrosion as a primary cause of pipeline ruptures, with an average occurrence period of 0.2 years, and external corrosion being the most prevalent form of damage [6, 7]. Bioreactor systems, especially those for bioleaching valuable elements from ores and electronic wastes, are known to suffer from MIC [8–10]. Chemical, mechanical and biological actions are main reasons behind corrosion behaviors in soil environment [11, 12]. Corrosion in soil environment has been widely studied due to the vast number of buried pipelines and tanks, as their deterioration can pose significant economic and environmental challenges over time [13–15].

Microbiologically influenced corrosion (MIC) is considered as a major contributor to external corrosion of pipes in soil [16, 17]. Various microorganisms such as bacteria, fungi, algae and archaea can lead to MIC [18–20]. Sewage sludge is abundant with nutrients and microbes, and it is often used as soil amendment to improve soil fertility and enhance the physical properties of soil [21, 22]. The increased concentrations of nutrients in soil from fertilizers, such as nitrates (NO_3_^−^) and ammonium (NH_4_^+^) have the potential to elevate corrosion rates by promoting microbial growth [23, 24]. Apart from aerobic microorganisms, anaerobic microorganisms such as sulfate reducing bacteria (SRB) can also grow in soil where oxygen diffusion is limited especially in deep layers [25].

SRB are believed to cause the most severe MIC among all corrosive microbes [26, 27]. Different mechanisms were proposed to explain the behavior of anaerobic SRB MIC. One is extracellular electron transfer MIC (EET-MIC) which mainly happens in SRB MIC of energetic metals such as iron [28, 29]. In EET-MIC, electrons released from extracellular metal oxidation are transported across cell walls to SRB cytoplasm for sulfate reduction to produce energy [30]. This behavior is explained by the biocatalytic cathodic sulfate reduction (BCSR) theory [28].

1 $${\text{Extracellular}}:{\text{ 4Fe}}\to {\text{4Fe}}^{{{2} + }} + {\text{ 8e}}^{ - } \left( {E^\circ \, = \, - {\text{447 mV}}_{{{\text{SHE}}}} } \right)$$

2 $${\text{Intracellular}}:{\text{ SO}}_{4}^{2 - } + \, 9{\text{H}}^{ + } + \, 8e^{ - } \to {\text{HS}}^{ - } + \, 4{\text{H}}_{2} {\text{O }}\left( {E^\circ{^\prime} \, = \, - 217{\text{ mV}}_{{{\text{SHE}}}} } \right)$$

EET promoters such as riboflavin was found to accelerate this type of MIC by accelerating the electron transfer process which will lead to higher MIC rates [31, 32]. Other bacteria such as nitrate reducing bacteria (NRB) were also reported to cause significant MIC [33, 34]. Facultative *Pseudomonas aeruginosa* can cause MIC both aerobically and anaerobically as NRB [35–39]. During the MIC of carbon steels and stainless steels by NRB *P. aeruginosa* biofilms, nitrate is employed as the terminal electron acceptors and reduced to ammonium or nitrogen gas [40],

3 $$2{\text{NO}}_{3}^{ - } + \, 10{\text{e}}^{ - } + \, 12{\text{H}}^{ + } \to {\text{ N}}_{2} + \, 6{\text{H}}_{2} {\text{O}} \left( {E^\circ{^\prime} \, = \, + 749{\text{ mV}}_{{{\text{SHE}}}} } \right)$$

4 $${\text{NO}}_{3}^{ - } + \, 8{\text{e}}^{ - } + \, 10{\text{H}}^{ + } \to {\text{ NH}}_{4}^{ + } + \, 3{\text{H}}_{2} {\text{O}} \left( {E^\circ{^\prime} \, = \, + 358{\text{ mV}}_{{{\text{SHE}}}} } \right)$$

Sewage sludge can be polluted by *P. aeruginosa* as well as other pathogenic microbes [41–43]. Normally, these pathogenic microbes are in low amounts. However, under certain environmental conditions, pathogenic microbes may grow to a level high enough that becomes a health risk [44, 45]. Antimicrobial agents such as biocides and antibiotics are commonly used to treat biofilms [46, 47]. Unlike planktonic cells, biofilms contain concentrated sessile microbes, which offer various defense mechanisms to the biofilms against environmental threats such as antimicrobials [48, 49]. Thus, biofilms often require 10X or higher concentrations of antimicrobials to treat compared with planktonic cells [50, 51].

Currently, all the biofilm assays or MIC test kits on the market are actually microbiology or molecular biology test kits which only monitor microbes [52–54]. They provide information on microbial activity, types or species, or the presence of important genes. However, they don’t provide biofilm corrosivity information. And a list of corrosive microbes from metagenomics analysis is of limited value without corrosion data. Besides, all these tests can hardly provide continuous data throughout the test period, and they are not convenient enough to give real-time biofilm or MIC rate information. In MIC studies, weight loss measurement and pitting analysis are two traditional and direct approaches to study corrosion. However, they require coupon tests which can only provide cumulative results at the end of the corrosion period. Therefore, a new convenient method has been desired for continuous monitoring of biofilms and MIC rates.

Electrochemical tests are also powerful tools widely used to provide extra corrosion information to support coupon weight loss results. Numerous MIC studies proved that electrochemical techniques such as linear polarization resistance (LPR) and Tafel were able to consistently support weight loss results and reflect corrosion rate trends correctly with additional transient insights [55–58]. Besides, they have an advantage over weight loss tests as electrochemical tests can provide transient corrosion data which makes near real-time MIC measurement possible. Furthermore, when the corrosion was not severe enough to generate measurable weight loss, electrochemical tests can be used as a sensitive tool to detect corrosion rates such as in titanium and stainless steel corrosion [59–61]. Therefore, a new electrochemical biofilm test kit based on electrochemical tests was developed to monitor biofilm health continuously and assess antimicrobial treatment of biofilms [62, 63]. The disposable biofilm/MIC test kit was based on a 2-electrode (2E) setup consisting of a working electrode (WE) and a counter electrode (CE) also serving as a pseudo-reference electrode (p-RE) while skipping a dedicated reference electrode which is fragile and not suitable for convenient use. Compared to the traditional 3-electrode (3E) setup in MIC measurements, the 2E scan did not yield the same corrosion rate numbers, but it was as accurate in correctly reflecting the corrosion rate trends [37, 63]. It was found to be reliable in correctly interpreting biofilm growth and antimicrobial treatment effect through changes in corrosion rates [37, 62]. But the biofilms to be assessed using this technique need to exhibit at least mild biocorrosivity, which is the limitation of the biofilm/MIC test kit.

In this work, a drainage ditch sludge was collected with metagenomics analysis conducted. To confirm the biocorrosivity of this sludge, X65 carbon steel coupons were used for weight loss measurement by simulating the worst-case scenario. Then, the newly invented electrochemical biofilm/MIC test kit which is sensitive in detecting MIC was adopted to monitor the biocorrosivity of the sludge biofilm [62]. The biofilm/MIC test kit was employed to assess semi-solid samples for the first time. The biofilm growth and the efficacy of a readily biodegradable biocide tetrakis-hydroxymethyl phosphonium sulfate (THPS) [64] in inhibiting biofilm growth were recorded and reflected by the variations in corrosion rates. The biocide injection test was also performed to assess the treatment effect of THPS on the pre-established sludge biofilm.

## Experimental

### Sludge, metal, and chemicals

Sludge samples (pH 6.8) were collected from the drainage ditch behind Stocker Center at Ohio University main campus (GPS coordinates 39.326839, − 82.107100) in July 2024. Sludge was sampled from sludge layers in the drainage ditch at three random locations and preserved together in a glass bottle. Sludge samples were evenly mixed with magnetic stirrer before tests to ensure consistency across replicates. Sludge Shotgun metagenomics analysis of the sludge sample was conducted before tests (service provided by SeqCenter, LLC, Pittsburgh, PA, USA). Various pathogenic microbes were found present in the sludge according to a metagenomics analysis. X65 carbon steel coupons were used in this test. Its elemental composition is shown in Table 1. An inert liquid Epoxy coating (3M Product 323) was used to protect all the surfaces of X65 coupons except for the top working surface (1 cm × 1 cm square). Before putting into the sludge, the coupon working surfaces were polished to 600 grit, sterilized with absolute isopropanol, and dried under UV light. To simulate the worst-case scenario, a concentrated nutrient solution was prepared to supply nutrients to the aerobic sludge to promote microbial growth. The composition of the concentrated nutrient solution is listed in Table 2. These nutrients are commonly added in industry such as wastewater treatment and food processing, which means they have the chance to be discharged into the environment such as soil and the drainage system [65–69]. The concentrations of these nutrients can promote the growth of corrosive microbes such as SRB to simulate the worst-case scenario [70].

**Table 1:** Elemental composition (wt. %) of X65 carbon steel [83].

Metal	C	Si	Mn	P	S	V	Nb	Ti	Fe
X65	0.16	0.45	1.60	0.015	0.006	0.06	0.04	0.04	Balance

**Table 2:** Composition of concentrated nutrient solution.

Chemical	Amount
Sodium lactate	170 g
Yeast extract	50 g
Sodium citrate	25 g
Fe(NH_4_)_2_(SO_4_)_2_·6H_2_O	68.5 g
DI water	1 L

### Biofilm visualization

Three replicate X65 coupons were incubated in a 10 mL vial containing 5 mL aerobic sludge for 7 d at 25 ℃. To make comparison, 100 ppm THPS was added into another set of vial with sludge and coupons. After that, coupons were taken out from the vials and rinsed with pH 7.4 phosphate buffered saline (PBS) solution to get rid of sludge and planktonic cells. A confocal laser scanning microscope (CLSM) (Model LSM 510, Carl Zeiss, Jena, Germany) was employed for biofilm observation. Biofilms on the coupon surfaces were first stained with Live/Dead® BacLight™ Bacterial Viability Kit L7012 (Life Technologies, Grand Island, NY, USA) [37]. Then, they were observed under CLSM where live cells were detected as green dots and dead as red dots.

### Weight loss and pitting observation

**Fig. 1:**
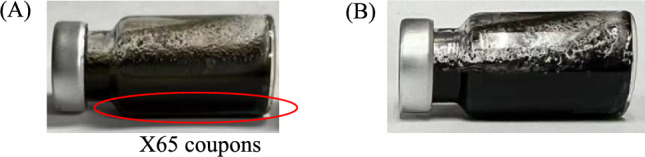
X65 coupons lined up in 10 mL vials before (**A**) and after (**B**) 7-d immersion in 5 mL sludge with nutrient enrichment without venting at 37 ℃

Three replicate X65 coupons were incubated in one 10 mL glass vial containing 5 mL aerobic sludge for 7 d at 25 ℃. Another three X65 coupons were incubated in a separate 10 mL glass vial (Fig. 1) containing 5 mL sludge with enrichment by adding 0.1 mL concentrated nutrient solution for 7 d at 37 ℃ without venting to promote the growth of corrosive anaerobic bacteria such as SRB to simulate the worst-case scenario. After 7 d, a fresh Clarke’s solution (1 L 37% hydrochloric acid (v/v) + 20 g antimony chloride + 50 g stannous chloride) was applied to clean coupons to remove biofilms and corrosion products from coupon surfaces following the ASTM G1–03 protocol. The weight of each coupon at 0 d and at 7 d was compared to obtain weight losses. X65 coupon surfaces were also scanned under an InfiniteFocus microscope (IFM) for pitting observation.

### Electrochemical measurements

**Fig. 2:**
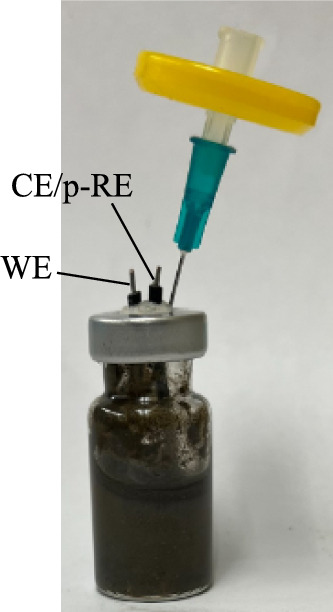
A 10 mL biofilm/MIC test kit vial consisting of an X65 WE and a graphite CE/p-RE filled with 5 mL aerobic sludge

Electrochemical tests were performed using an electrochemical biofilm/MIC test kit (Fig. 2) consisting of an X65 WE and a graphite rod (0.64 cm diameter and 1 cm height) CE/p-RE in a 10 mL serum vial [63]. A 0.22 μm membrane filter was connected for venting to maintain aerobic condition. Electrochemical scans were conducted using a PCI4/750 potentiostat (Gamry Instruments, Inc., Warminster, PA, USA). LPR and Tafel were scanned on a daily basis within a 7-d period. LPR was measured with a scan rate of 0.167 mV/s within the voltage range of −10 to 10 mV vs. OCP (open circuit potential). Tafel was scanned at a rate of 0.167 mV/s on the same WE between OCP − 200 mV and OCP + 200 mV using dual-half scans starting from OCP [71]. This dual-scan method is rather gentle and allows repeated Tafel scans on the same WE without causing damage on it [71]. Another scan method named continuous upward scan (directly from OCP − 200 mV to OCP + 200 mV) was also conducted once on the same X65 WE at the end to compare the obtained curve with the one from dual-half scans [71].

## Results and discussion

### Metagenomics

**Fig. 3:**
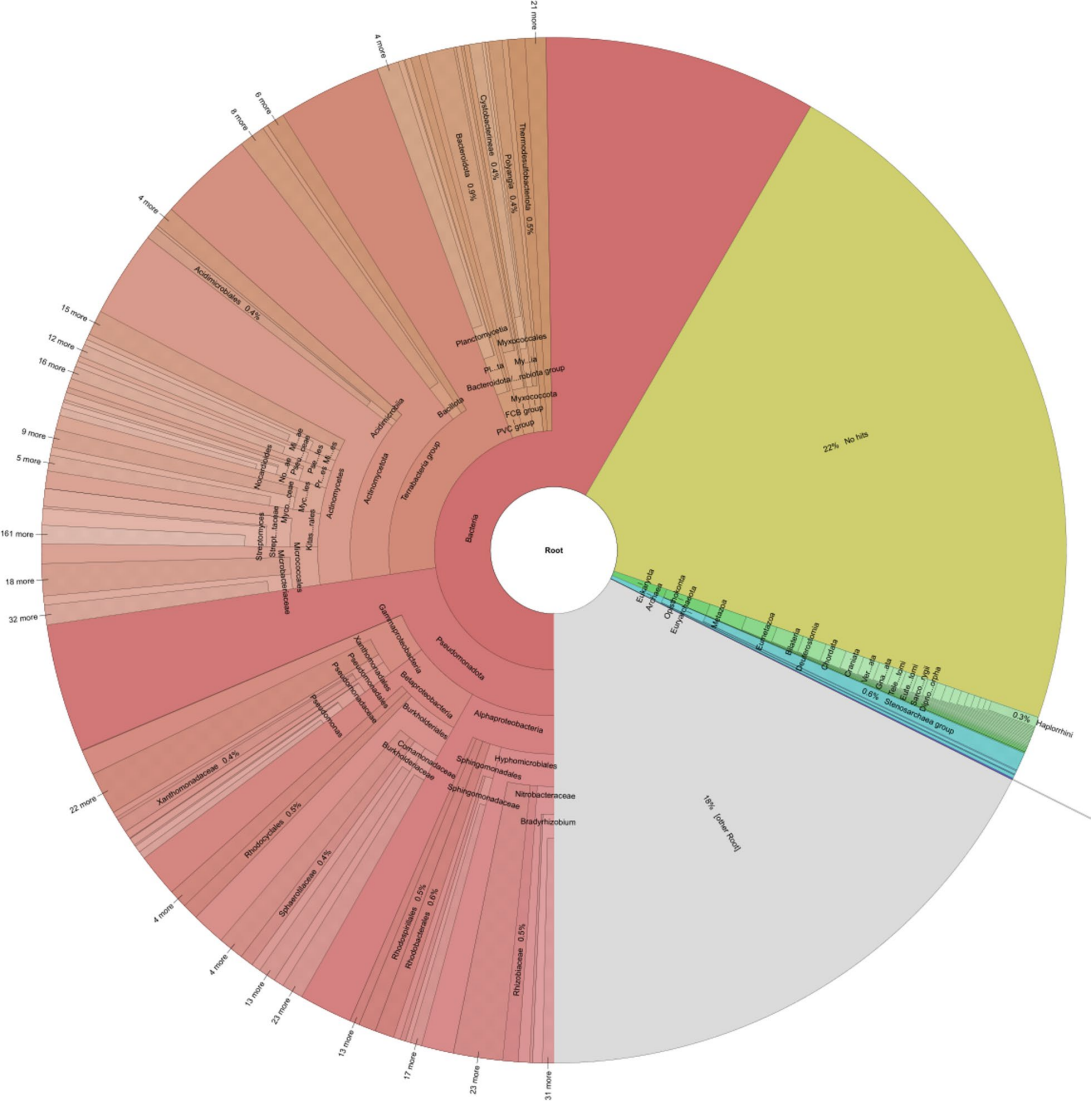
Metagenomics of sludge sample

The metagenomics analysis of the sludge sample indicates that 1.2% of various SRB were detected among the Thermodesulfobacteriota, Bacillota, etc. phyla which can be found in Fig. 3. Although aerobic sludge was not ideal for anaerobic SRB growth, the limited access of the bottom layer of the sludge to oxygen in test kit vials can provide anaerobic conditions for SRB to grow. Besides, 0.05% *P. aeruginosa* was also detected. These bacteria were known to be capable of causing MIC. Although these corrosive bacteria are not dominant microbes in the sludge samples, they are able to cause MIC especially EET-MIC since only the bottom layer of a synergistic mixed-culture biofilm on the metal surface is involved in EET due to the distance limit in electron transfer [29]. It is common that a consortium biofilm containing a small percentage of corrosive species can lead to severe MIC [72]. Therefore, the metagenomics results suggest the potential of the sludge to cause MIC.

### Biofilm observation

**Fig. 4:**
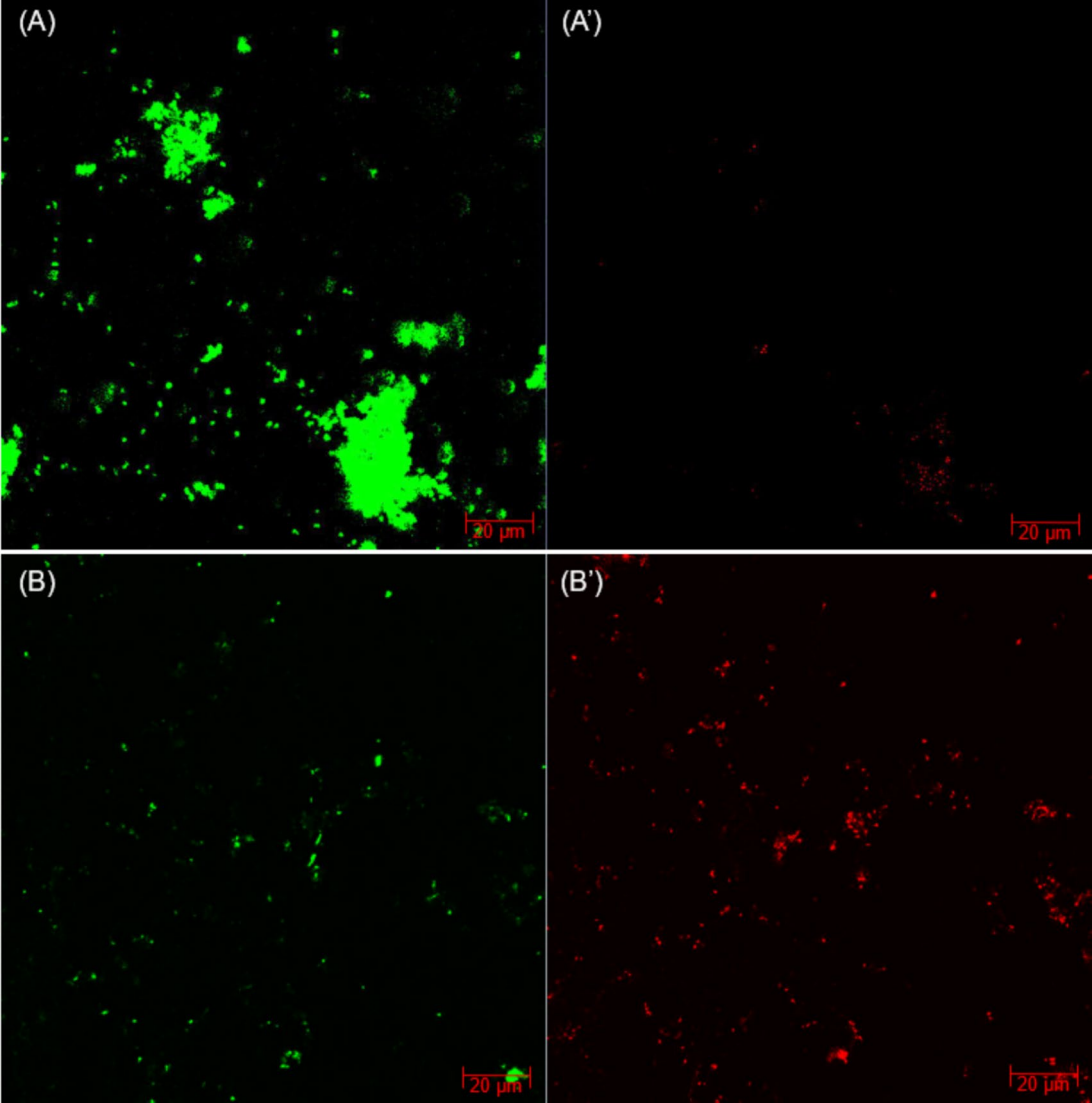
CLSM images of aerobic sludge biofilms on X65 coupons after 7 d at 25 ℃ with aerobic sludge with no treatment (**A**, A’), and 100 ppm THPS (**B**, B’). A and B show live cells only. A’ and B’ show dead cells only

Figure 4 demonstrates 7-d aerobic sludge biofilms on the X65 coupon surfaces. Without biocide, healthy biofilms were formed on the coupon surface with abundant live cells (green dots) in the biofilm. A few dead cells (red dots) were also detected which might be aerobic microbes deep in sludge that were not able to access sufficient oxygen. In comparison, with 100 ppm THPS addition at the very beginning, the number of live cells were considerably reduced while many more dead cells were observed in the biofilms. Thus, the biocidal effect of THPS was manifested. The CLSM results prove that the sludge was able to generate biofilms on the X65 coupon surface with sufficient live cells to cause potential MIC. And THPS was effective in preventing the sludge biofilm formation as in preventing other SRB and NRB biofilm growth [37, 73].

### Weight loss and pitting analysis

**Fig. 5:**
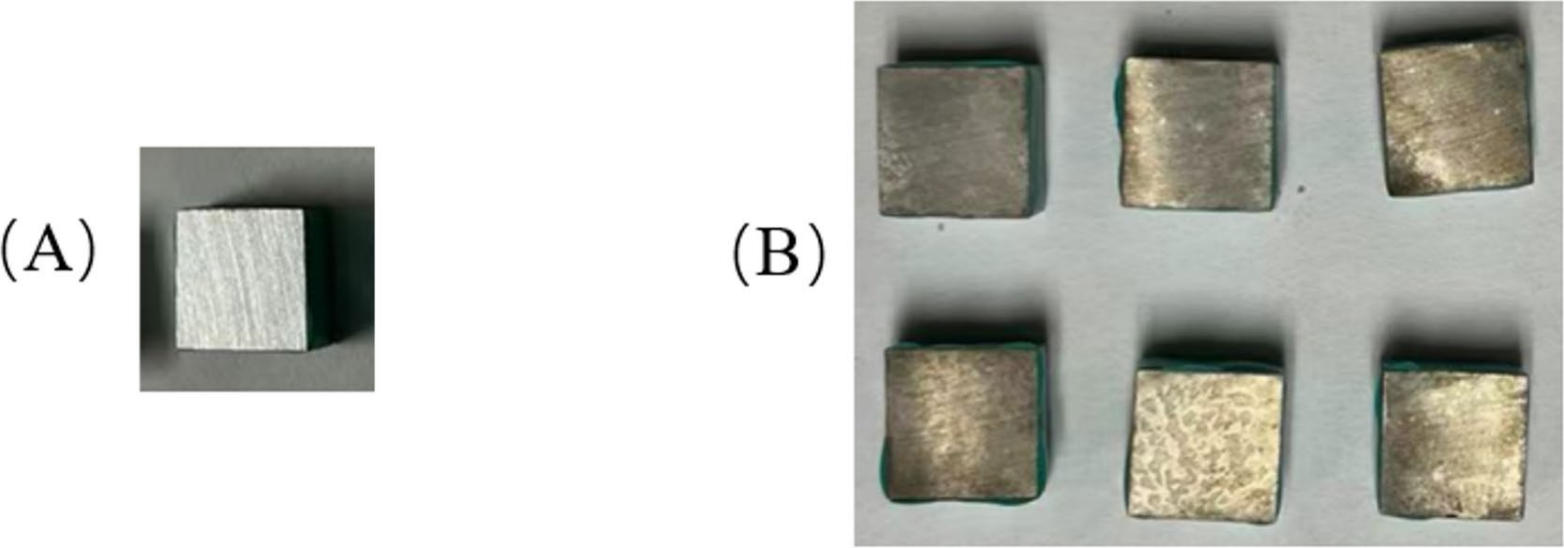
**A** an X65 coupon before immersion in sludge, and (**B**) X65 coupons after 7 d in sludge and cleaned with Clarke’s solution (coupons in first row were in aerobic sludge at 25 ℃ and coupons in second row were in sludge with nutrient enrichment without venting at 37 ℃)

**Fig. 6:**
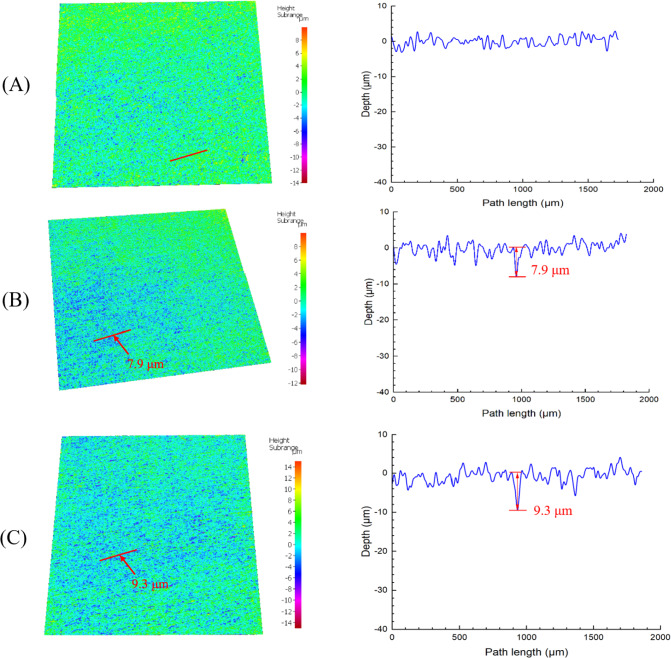
IFM pit images and pit profiles of (**A**) pristine X65 coupon, and X65 coupons after 7-d immersion in (**B**) aerobic sludge at 25 ℃, and (**C**) sludge with nutrient enrichment at 37 ℃

After 7 d, coupons in sludge all showed discoloration compared to pristine coupons as shown in Fig. 5, suggesting potential MIC by the sludge. Uniform corrosion and pitting corrosion are two main categories of MIC. Uniform corrosion is commonly evaluated by weight loss measurement. However, after 7 d, X65 coupons in aerobic sludge did not yield meaningful weight loss (0.0 ± 0.1 mg/cm^2^), indicating that the corrosivity of the biofilm was small. Thus, pitting analysis was performed to check corrosion evidence. It is a common phenomenon in MIC studies when the biofilms are not corrosive enough or the metals such as titanium and stainless steels are too corrosion-resistant to generate measurable weight losses [60, 61, 74]. In Fig. 6, the pristine X65 coupon did not show well-defined pits. In comparison, the X65 coupon in aerobic sludge had a much rougher surface profile with a maximum pit depth of 7.9 μm (0.41 mm/a). The rough surface was a result of uniform corrosion by the sludge biofilm although it was not severe enough to generate measurable weight loss. The pitting corrosion on the X65 coupon proves the biocorrosivity of the sludge biofilm, which is similar to other cases with only mildly corrosive biofilms so that weight loss was very small or even undetectable while the corrosion mainly manifested in the form of pitting corrosion [74, 75]. The pitting corrosion was also found to be much smaller compared to other SRB/NRB MIC cases with low corrosivity [30, 76].

To simulate the worst-case scenario when additional nutrients are available, and to investigate the potential of the sludge to cause significant uniform corrosion, 0.1 mL concentrated nutrient solution (composition in Table 2) were added in one separate vial containing 5 mL sludge at the very beginning without venting at a higher temperature to promote the growth of corrosive anaerobic microbes such as SRB and NRB after O_2_ depletion. As expected, the nutrient enrichment improved the biocorrosivity of the sludge and resulted in a (0.5 ± 0.1) mg/cm^2^ weight loss (0.03 mm/a uniform corrosion rate) on X65 coupons. The 7-d weight loss is smaller than that on carbon steels caused by SRB *Desulfovibrio vulgaris* (2.5 mg/cm^2^) [31] and mildly-corrosive NRB *Bacillus licheniformis* (0.89 mg/cm^2^) [30]. However, compared to the aerobic sludge without enrichment case, the potential of the sludge to cause significant uniform corrosion under nutrient contamination conditions was manifested. During summertime, pipelines buried in soil in contact with sludge may suffer from uniform corrosion. In Fig. 6C, a 9.3 μm pit (0.48 mm/a) was found on the X65 coupon surface. Compared to the aerobic sludge case, nutrient enrichment increased the maximum pit depth by 18%, suggesting more severe pitting corrosion. The pitting capability of the sludge was not considerably increased compared to the improvement in the uniform corrosion, which was attributed to the fact that pitting corrosion is not as dominant as uniform corrosion in MIC of carbon steels [77].

### Electrochemical measurements

**Fig. 7:**
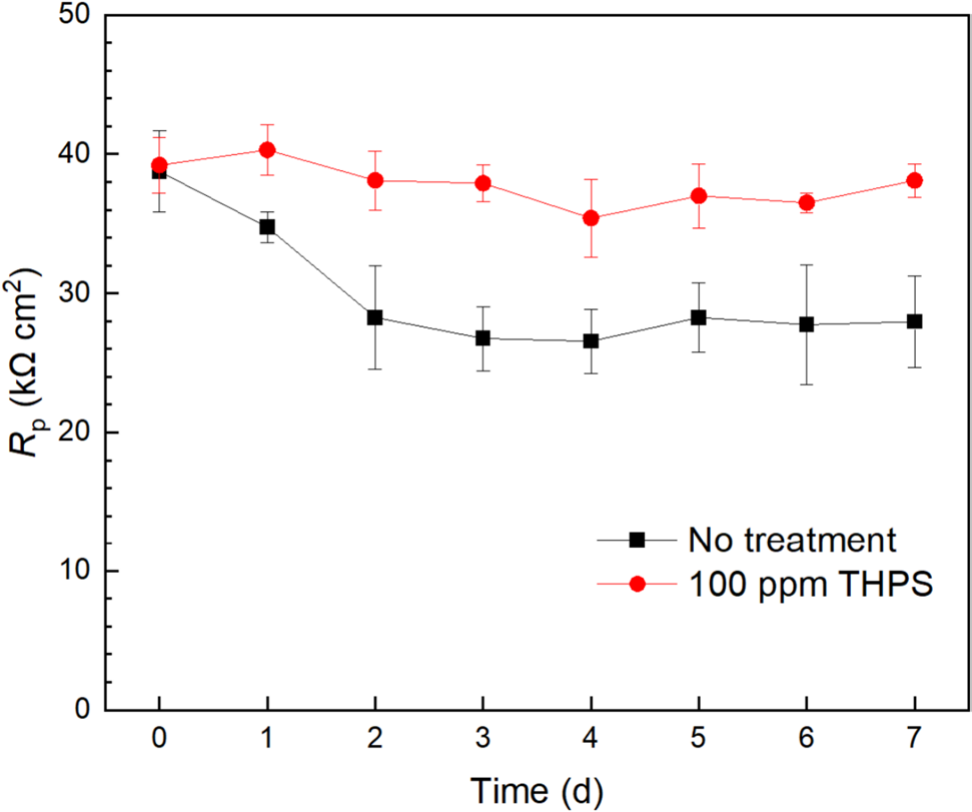
LPR *R*_p_ curves of X65 WEs during 7-d immersion at 25 ℃ in 10 mL test kit vials each containing 5 mL aerobic sludge. (Range bars obtained from two replicate test kit vials)

Electrochemical test results can contribute more transient corrosion information throughout the entire corrosion period compared to the one-shot weight loss or pitting analysis. Figure 7 displays *R*_p_ (polarization resistance) curves from LPR scan of X65 WEs in aerobic sludge. Without biocide, the corrosion rate of the sludge biofilm (reflected by 1/*R*_p_) generally increased from 0 d to 3 d, corresponding to biofilm growth on the X65 WE surface. The *R*_p_ curve levelled off for the remaining days. The *R*_p_ results correspond to a typical MIC trend [62], confirming the biocorrosivity of the sludge biofilm. In comparison, with the 100 ppm THPS added at the very beginning, the *R*_p_ curve only showed a very slight decrease, suggesting no or very small corrosivity because the sludge biofilm growth was inhibited by THPS. The disulfide bonds in disulfide amino acids within microbial cell walls can be effectively reduced by trihydroxymethyl phosphine degraded from THPS [78, 79]. This process leads to bond cleavage and subsequent cell wall destruction. Additionally, THPS selectively impacts the activities of heterotrophic nitrate-reducing bacteria (hNRB) and sulfide-oxidizing NRB (so-NRB), thereby inhibiting SRB growth and preventing sulfide production [80].

**Fig. 8:**
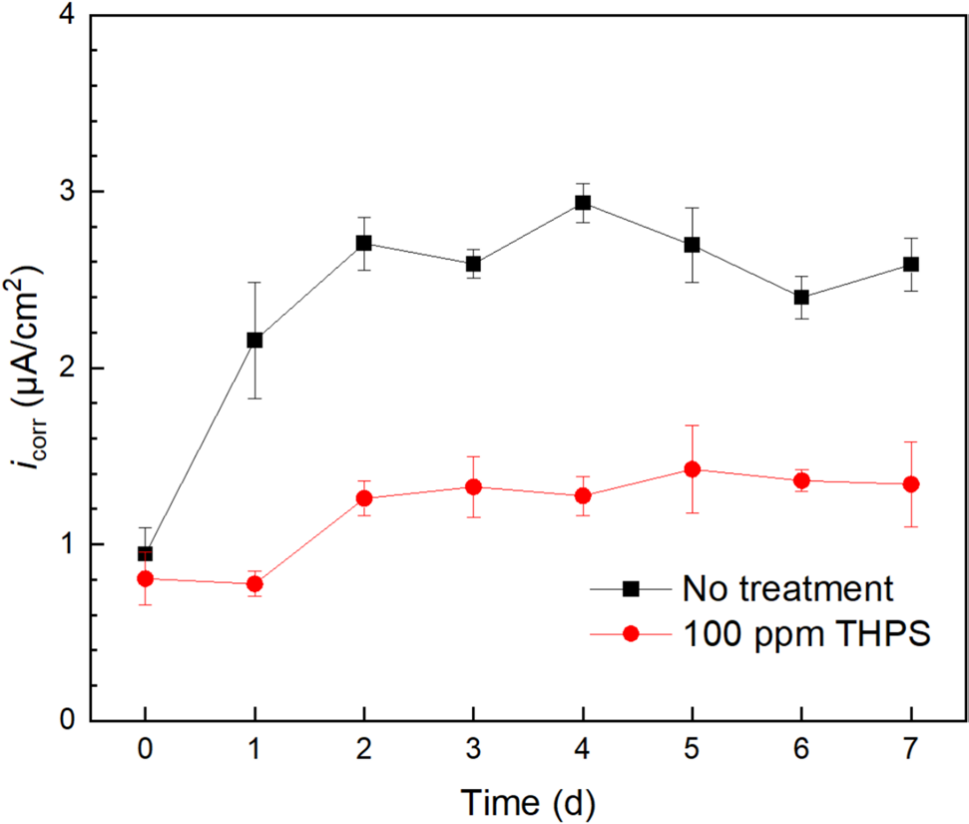
*i*_corr_ curves of X65 WEs from Tafel scans during 7-d immersion at 25 ℃ in 10 mL test kit vials each containing 5 mL aerobic sludge

Tafel scan results were consistent with LPR findings. In Fig. 8, the corrosion current density (*i*_corr_) curves based on Tafel scan data demonstrate the same corrosion rate trend as *R*_p_ curves. The corrosivity of the sludge biofilm without biocide treatment generally increased with biofilm formation and levelled off after biofilm maturity. In comparison, with 100 ppm THPS, the increase in corrosion rate was much smaller, showing the biofilm prevention effect of THPS. Both LPR and Tafel scans supported the previous biofilm observation results. Therefore, the two electrochemical test results conducted in the biofilm/MIC test kit were able to correctly monitor biofilm growth, biofilm health and biocide treatment effect. In both LPR and Tafel tests, the 100 ppm THPS was found effective in preventing MIC caused by the sludge biofilm, which is consistent with findings in other studies that 100 ppm THPS successfully inhibited the growth of corrosive SRB [73, 81]. Thus, THPS was proved to be effective in inhibiting the growth of the aerobic sludge biofilm.

**Fig. 9:**
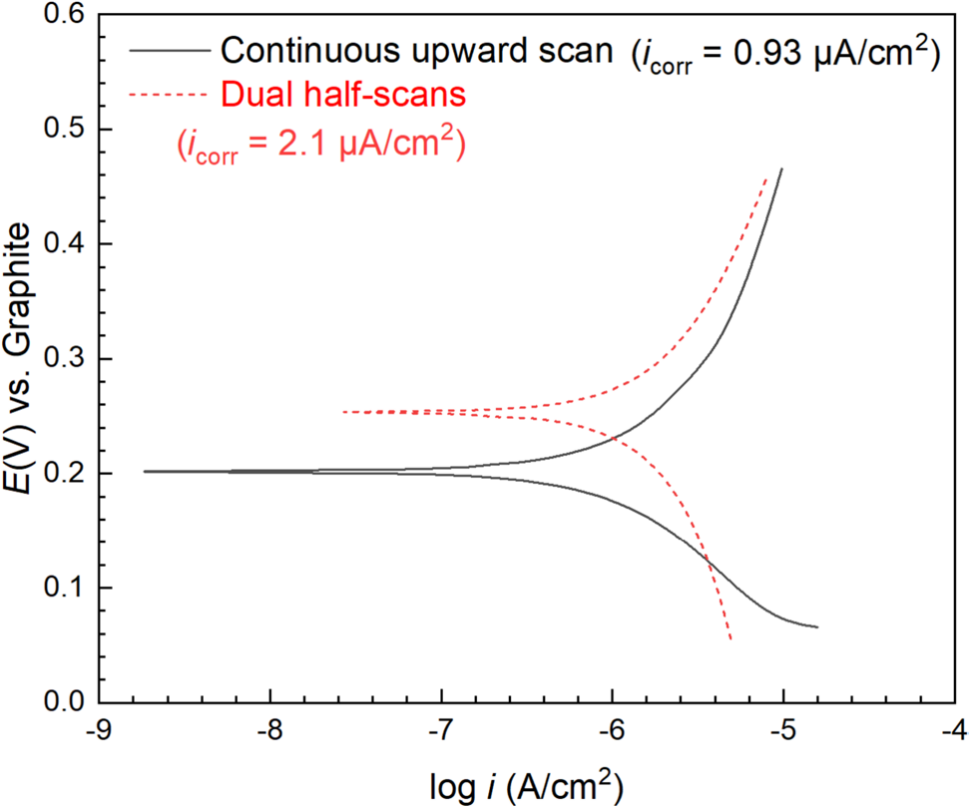
Tafel curves at 7 d obtained using different scan methods on the same X65 WE in 10 mL test kit vial containing 5 mL aerobic sludge at 25 ℃

Figure 9 displays Tafel curves obtained using two different scan methods on the same X65 WE in aerobic sludge without biocide at 7 d. It’s obvious that the Tafel curve obtained by dual-half scans exhibit a relatively symmetric shape with both anodic and cathodic regions covering 200 mV. However, the continuous upward scan generated a skewed Tafel curve as the cathodic region is obviously compressed and the anodic region elongated. Compared to the dual-half scan curve, the *E*_corr_ obtained by continuous upward scan also deviated from 254 mV to 201 mV (vs. graphite RE). Besides, the *i*_corr_ value from continuous upward scan (0.93 μA/cm^2^) was also 56% smaller than that from dual-half scans (2.1 μA/cm^2^). This Tafel skew phenomenon suggests the existence of MIC caused by biofilms on the WE as the continuous upward did not allow sufficient time for the biofilm to adapt to the extreme external voltage [63, 71]. Thus, the Tafel curves also confirm the corrosivity of the sludge biofilm.

**Fig. 10:**
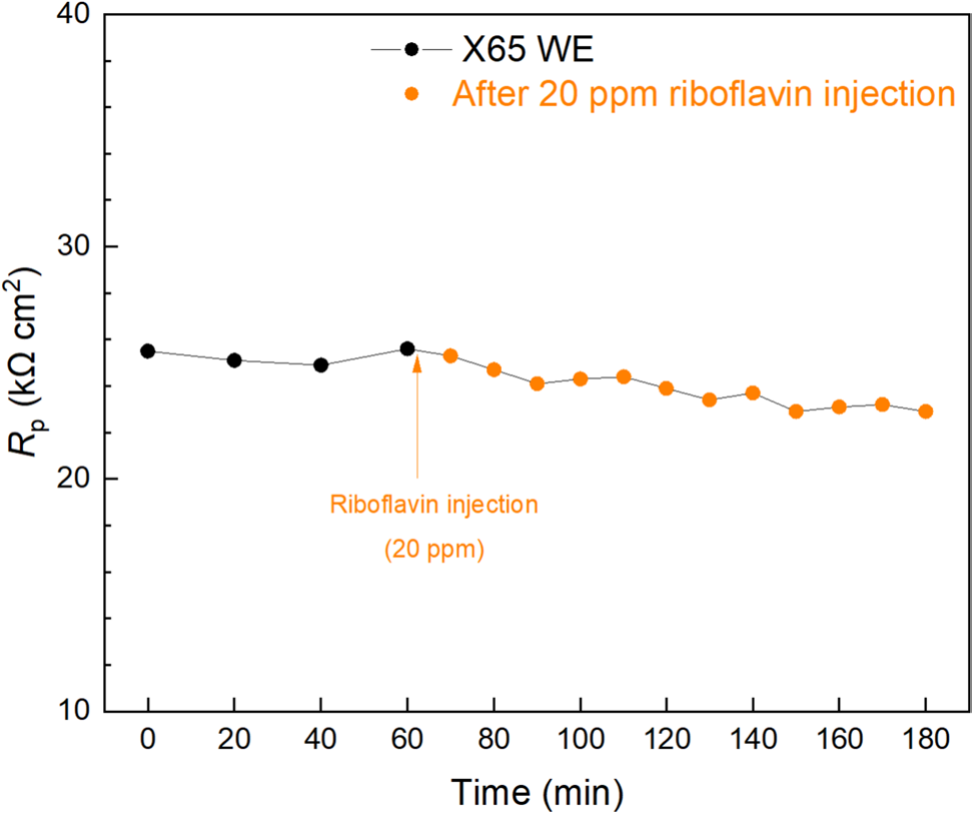
*R*_p_ response of X65 WE to riboflavin (EET promoter) injection at 7 d in a 10 mL test kit vial containing 5 mL aerobic sludge. (Time zero was at 7 d of immersion prior to injection)

**Fig. 11:**
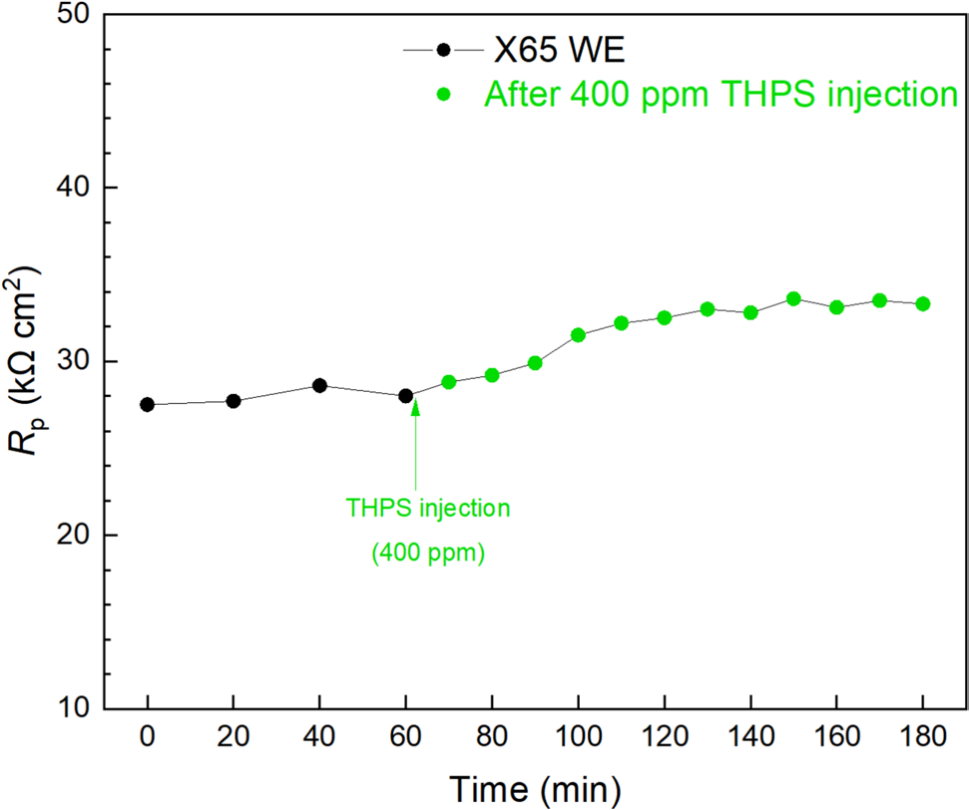
*R*_p_ response of X65 WE to THPS injection at 7 d in a 10 mL test kit vial containing 5 mL aerobic sludge

Injection tests were conducted in biofilm/MIC test kit vials at 7 d to monitor the responses of mature biofilms to chemical injections. In the abiotic corrosion systems, the injections of EET promoters (e.g. riboflavin) and biocide will have negligible impact on the *R*_p_ curves [63]. In Fig. 10, a stable *R*_p_ curve was first obtained within 1 h, after which 20 ppm riboflavin injection was performed. The *R*_p_ value was then generally decreased (corrosion rate increased) over time and stabilized around 2 h with an overall 10% *R*_p_ drop (a 3-d injection test also showed similar trend in Fig. S1). This indicates the presence of EET-MIC by SRB or NRB in the sludge biofilm which can be accelerated by EET promoters such as riboflavin [31]. Another injection test using 400 ppm THPS biocide was conducted in another test kit vial. In the previous biofilm prevention test, 100 ppm THPS was selected and found effective in inhibiting biofilm growth. However, treating an established biofilm such as at 7-d of incubation is far more difficult than in the prevention test due to various defense mechanisms possessed by biofilms [82]. Thus, a much higher dosage is usually needed to treat a mature biofilm. After the 400 ppm THPS injection, the *R*_p_ was increased (corrosion rate decreased) by 21% as shown in Fig. 11 (a 3-d injection test also showed similar trend in Fig. S2), which suggests the effectiveness of THPS in killing pre-established sludge biofilm. The transient changes in sludge biofilm corrosion rates were accurately reflected by the electrochemical data from the biofilm/MIC test kit. This provides the test kit an edge over other one-shot tests which cannot catch transient information. The percentages in *R*_p_ decrease and increase due to riboflavin and THPS was not as great as in other MIC systems [37, 74]. It also took a longer time for the *R*_p_ curves to become stabilized. This can be attributed to the slower diffusion in semi-solid sludge compared to pure liquid, which made chemicals more difficult to reach biofilms. The mild corrosivity of the aerobic sludge may also explain the small *R*_p_ decrease and increase as the potentials of the corrosivity to be improved or reduced were limited.

### Further consideration

This study was conducted in a static condition simulating external corrosion of buried pipeline that get in contact with semi-solid corrosive agents such as sludge. To better simulate the real pipeline environment, a flow system will be employed to evaluate the effects of flows on the sludge biofilm formation and corrosion rate changes. Besides, a long-term test (e.g., more than one month) needs to be performed to monitor the corrosion behavior of matured biofilms to see whether the corrosion will be accelerated or decelerated after weeks due to changes in temperature, nutrient concentrations, etc. A more comprehensive metagenomics study will also be performed to explore the corrosion mechanisms. Different biocides such as glutaraldehyde and 2,2-dibromo-3-nitrilopropionamide (DBNPA) can be tested apart from THPS to investigate their efficacy in mitigating MIC caused by sludge biofilms. This work confirmed the capability of the biofilm/MIC test kit in assessing a semi-solid sludge sample for the first time. Testing other semi-solid samples is also necessary to prove the applications of the biofilm/MIC test kit in analyzing samples of different forms.

## Conclusion

This work confirmed the biocorrosivity of the aerobic sludge against X65 carbon steel. The corrosivity of the aerobic sludge biofilm without nutrient enrichment did not produce measurable uniform corrosion on carbon steel in 7 d, but pitting corrosion was observed. After enrichment with nutrients, the sludge at a higher temperature without venting led to (0.5 ± 0.1) mg/cm^2^ weight loss (0.03 mm/a uniform corrosion rate) and more severe pitting corrosion on X65 carbon steels, suggesting the potential of the sludge to cause significant uniform corrosion under nutrient contamination conditions.

The biocorrosivity of the sludge biofilm was correlated with its biofilm growth and health on X65 working electrodes in the biofilm/MIC test kit. The test kit successfully monitored sludge biofilm formation and biofilm inhibition effect of THPS using LPR and Tafel scans. The biofilm/MIC test kit was capable of detecting mild pitting. The 100 ppm THPS was effective in preventing sludge biofilm growth, and 400 ppm THPS was needed to kill the pre-established sludge biofilm on X65. The corrosion rate of the sludge biofilm was accelerated by riboflavin (an EET promoter), suggesting the presence of EET-MIC. The disposable biofilm/MIC test kit was able to conveniently and easily monitor the biofilm health and its biocide treatment continuously. Therefore, it has the potential as a useful tool in assessing other corrosive biofilms and their biocide treatment efficacy. For future work, the biofilm/test kit will be used to assess sludge biofilms in different conditions such as in flow systems, in long-term and with different biocides. Other semi-samples will also be used for testing in the biofilm/test kit.

## Supplementary Information

Below is the link to the electronic supplementary material.

Supplementary file1 (DOCX 165 kb)

## Data Availability

No datasets were generated or analysed during the current study.
