# Supplementary material for: Assessing the performance of a disposable electrochemical biofilm test kit on monitoring drainage sludge biofilm corrosion and its biocide treatment
Source: Bioprocess Biosyst Eng. 2025 May 5;48(8):1255–66. doi: 10.1007/s00449-025-03173-x (PMC12234593; doi:10.1007/s00449-025-03173-x)
Supplement: Supplementary file 1 — Supplementary file1 (DOCX 165 kb) [file 449_2025_3173_MOESM1_ESM.docx]

**Assessing the performance of a disposable electrochemical biofilm test kit on monitoring drainage sludge biofilm corrosion and its biocide treatment**

Lingjun Xu^1^, Chris Gu^2^, Shaohua Wang^2,3,*^

^1^Department of Chemical & Biomolecular Engineering, Institute for Corrosion and Multiphase Technology, Ohio University, Athens 45701, USA

^2^Department of Biomedical Sciences, Ohio University Heritage College of Osteopathic Medicine, Ohio University, Athens, OH 45701, USA

^3^Infectious and Tropical Disease Institute, Ohio University, Athens, OH, 45701, USA

^*^Corresponding author: E-mail address: [wangs4@ohio.edu](mailto:wangs4@ohio.edu)

**Supplementary Materials**

Figs. S1 and S2 present polarization resistance (*R*_p_) curves of X65 woring electrodes (WEs) after injections of 20 ppm riboflavin and 400 ppm THPS tetrakis hydroxymethyl phosphonium sulfate (THPS) biocide at 3 d of incubation in two 10 mL electrochemical glass cells each containing 5 mL aerobic sludge. Around 2 h after the injections, the *R*_p_ value was decreased (corrosion rate increased) by 14% and increased (corrosion rate decreased) by 19%, respectively.


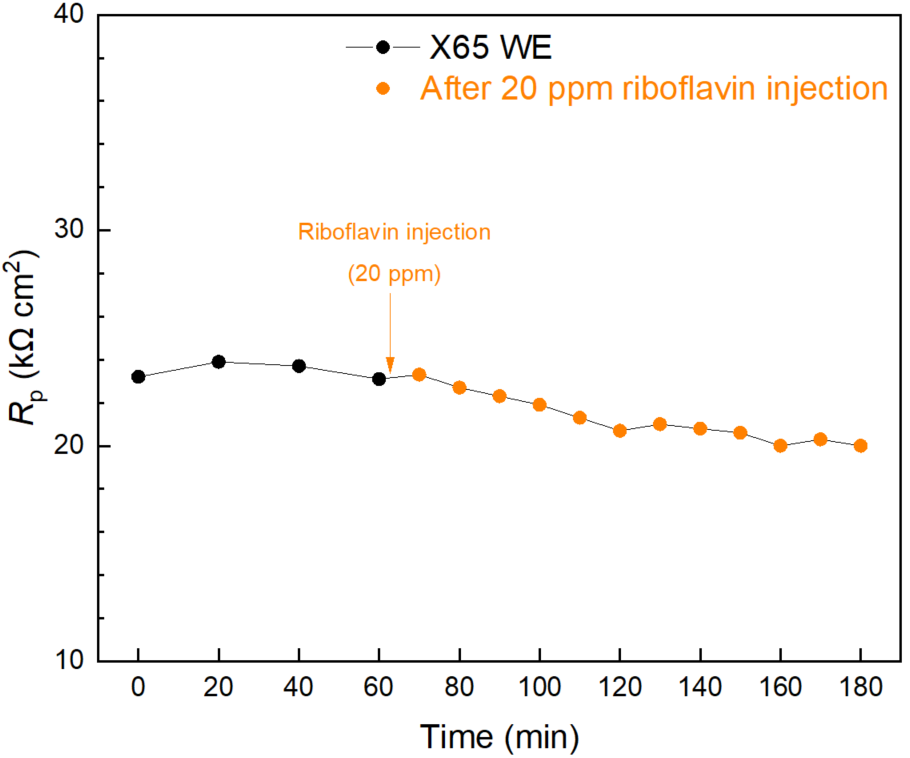


Fig. S1. *R*_p_ response of X65 WE to riboflavin injection at 3 d in a 10 mL test kit vial containing 5 mL aerobic sludge. (Time zero was at 3 d of immersion prior to injection.)


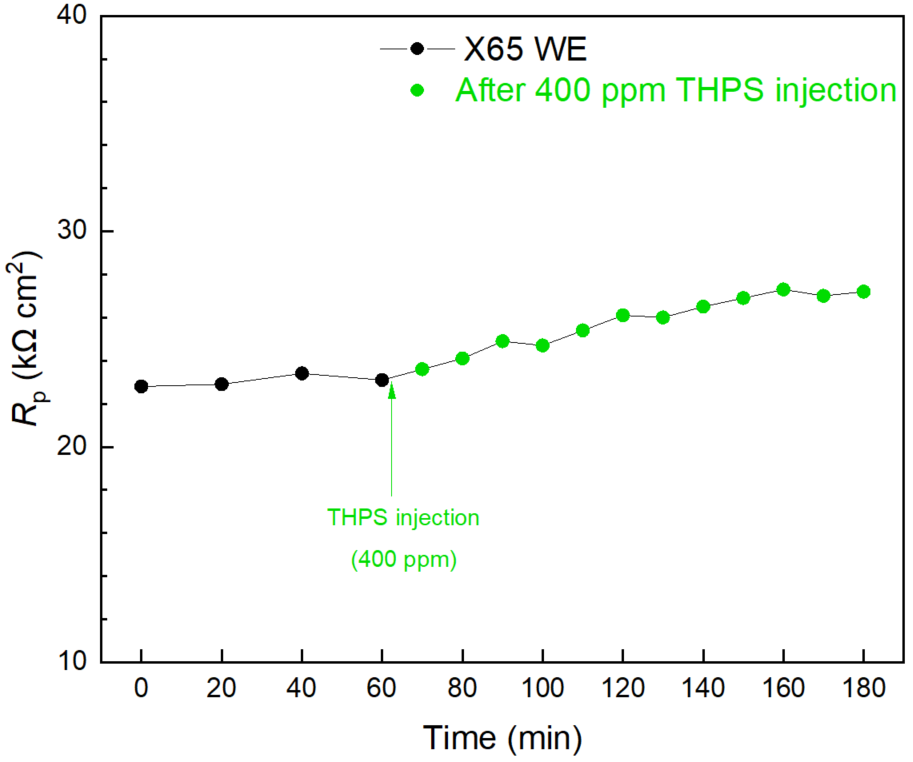


Fig. S2. *R*_p_ response of X65 WE to THPS injection at 3 d in a 10 mL test kit vial containing 5 mL aerobic sludge.
